# Driven Wells

**Published:** 1883-04

**Authors:** 


### DRIVEN WELLS.

GOOD drinking water is just as essential to health as pure air; but it is not always easy to determine if water is pure through the sense of taste or sight; water that contains considerable organic matter is sometimes offensive to the sense of smell; but this is not always the case. The best method of testing for organic impurities is to drop into a tumbler of the water just enough "permanganate of potash," or of "Condy's fluid," to give the water a pinkish color. Any organic matter contained in the water is precipitated after standing an hour, the water losing its color or becoming brownish. If, however, there are no impurities to be acted upon, the water remains of a pinkish color for hours. This is a very simple test, and a valuable one, and were it more frequently employed, and its indications heeded, there would be fewer cases of fever. The best plan to get pure drinking water is by means of what is generally known as the "driven well," but which by our plan is nothing of the sort. We have seen an open well, the water of which was dangerous to health, within ten feet of a "driven well," in the water of which no impurities could be detected. It is almost impossible to find an open well of perfectly pure water, and it is hardly possible to find a driven well where the water contains impurities.

The easiest way to make a "driven well," is to get a piece of iron or steel pipe, six feet long and one and a quarter inches in diameter; make a cutting edge at one end by beveling off inside the end of pipe, with a half round file, drive this pipe down a foot or two and then draw it up, and the earth from the hole will be found inside the pipe, and can be pushed out with an iron rod. Repeat this operation until you have gone six feet; then couple an inch pipe, six feet long, to your cutting pipe; continue this till water is reached, then attach a short piece of pipe having small holes in it, and a plug in the lower end, to your inch pipe, and drop it into the little well you have *dug*; screw on a pump and you have a well complete at about the price Mr. Green charges for the right to drive a pipe into the ground. In the decision of the Court in regard to the Green patent, the judge said, that where the earth was raised up and taken out of the well, it did not infringe Green's patent, which holds good only where a pipe is driven down so as to push the earth aside. But everybody knew that.

It has always seemed remarkable that any one should have bothered with Green's patent, when the plan above described is in all respects preferable and is hampered by no patent. Though small it is just as much an open well as if it were six feet in diameter, and any one has the undoubted right to construct such wells. If stones are encountered they may be broken and penetrated by attaching a piece of round steel, one inch in diameter and two feet long, to the end of the inch pipe. The lower end should be drawn down to a blunt point. We constructed a well on the above plan, using an inch galvanized pipe, and the supply of water is abundant for twenty families.

